# Valorization of *Arnica montana* Wastes after Extraction of the Ethanol Tincture: Application in Polymer-Based Matrices

**DOI:** 10.3390/polym13183121

**Published:** 2021-09-16

**Authors:** Noelia Flórez-Fernández, Tania Ferreira-Anta, María Dolores Torres, Herminia Domínguez

**Affiliations:** 1 Departamento de Enxeñería Quimica, Campus Ourense, Universidade de Vigo, Edificio Politécnico, As Lagoas, 32004 Ourense, Spain; noelia.florez@uvigo.es (N.F.-F.); ta.ferreiraan@gmail.com (T.F.-A.); herminia@uvigo.es (H.D.); 2 CINBIO, Departamento de Ingeniería Química, Campus Ourense, Universidade de Vigo, 32004 Ourense, Spain

**Keywords:** intensified extraction, antiradical capacity, antiproliferative, polymer-based gelled matrices, rheology

## Abstract

The waste solids remaining after the ethanolic extraction of arnica were proposed as raw material for the recovery of the remaining phenolic fraction. Greener processes based on intensification extraction, with microwave (MHG) and ultrasound (UAE) assistance and pressurized hot water extraction under subcritical conditions (AH), were studied. The entire process provided approximately 28% of phenolics for the sequence when MHG was used, 22% in the sequence where AH was employed, and the extracts showed up to 60% the ABTS radical scavenging capacity of Trolox. However, the cytotoxic effects on the cell growth of tumoral cells were only moderate. Therefore, considering a possible external topical application, extracts containing selected arnica extracts were further used to develop polymer-based gelled matrices formulated with either chitosan or polyvinyl alcohol. Rheological testing indicated that all proposed matrices exhibited relatively high mechanical features, even better than those determined with matrices prepared with their counterpart commercial arnica tinctures. Overall, the shear-thinning behavior of gelled matrices loaded with arnica extracts obtained by MHG or AH stages was enhanced compared to those containing ethanolic extracts, whereas the viscoelastic features became smoother for polymeric matrices incorporated with arnica extracts recovered at the highest MHG irradiation powers or at the highest set point temperatures of AH treatments.

## 1. Introduction

*Arnica montana* L. is a traditional medicinal plant and its flowers are used to alleviate pain and treat skin disorders for due to the fact of its tissue regeneration and healing capacity properties. The extracts or the different parts of the plant also have antioxidant, anti-inflammatory, antimicrobial, immunomodulatory, antitumor, antitussive, and bronchodilatory properties [1,2,3,4,5,6]. The flowers are the most used parts, and they contain sesquiterpene lactones, helenalin, and dihydrohelenalin and their esters, phenolic acids, lignans and flavonoids, carotenoids, essential oils, terpenoids, polysaccharides, and pyrrolizidine alkaloids [4]. The standardized flower extracts contain 4–14 mg/g sesquiterpene lactones and 9–18 mg/g of two major flavonoids: quercetin and kaempferol glucosides [7,8]. Values in the range 0.60–2.88% were reported for flavonoids and 0.96–2.24% for phenolic acids. Quercetin 3-O-glucuronic acid and kaempferol-3-O-glucoside were the dominant flavonoids, but quercetin 3-O-β-D-glucoside, patuletin 3-O-β-D-glucoside, kaempferol 3-O-β-D-glucuronide, 6-methoxykaempferol 3-O-β-D-glucoside, hispidulin, kaempferol, quercetin, apigenin, and rutine were also found. Protocatechuic and 3,5-dicaffeoylquinic acid were the major phenolic acids, but chlorogenic, caffeic, 1-methoxy-oxaloyl-3,5-dicaffeoylquinic acid, and 4,5-dicaffeoylquinic acid were also present [4,7,8,9,10,11,12,13]. The acetone exudates of flower heads were richer in surface flavonoids than leaf extracts and presented high contents of the flavonoids 6-OH luteolin 6-methyl ether and kaempferol-6-methyl ether.

Although the main anti-inflammatory and analgesic activity of arnica is due to the sesquiterpene lactones helenalin and 11, 13-dihydrohelenalinester, the flavonoid and phenolic fractions could also be of interest because their additional antioxidant and antimicrobial effects could act synergistically with sesquiterpene lactones [7,12,14]. Other authors have reported that a methanol extract alleviated arthritis in rats with better therapeutic efficacy than dexamethasone, and this action was probably due to the combined action of phenolic and flavonoid compounds [3]. The phenolic acid-rich extract from flowers showed high antioxidant capacity and lowered blood platelet aggregation without cytotoxicity [15]. Ethanolic extracts, rich in flavonoids and phenolic acids, showed good antioxidant activity and cytoprotective effects against hydrogen peroxide-induced oxidative damage in fibroblast-like cells [16]. *Arnica montana* is also rich in biologically active acidic polysaccharides and their glycoconjugates, which possess anticoagulant, antioxidant, anti-inflammatory and antitussive effects without any cytotoxicity effect in model cells [2].

Traditionally, arnica extracts are not recommended for oral administration due to the side effects [17]. More recently, different encapsulation techniques have proved suitable for the application of these extracts. Gaspar and coauthors reported the topical treatment of inflammation with liposomal formulations of polyphenols and polysaccharides, and Oliveira and coauthors incorporated arnica tinctures with other antimicrobials in biocompatible materials, such as polyvinyl alcohol (PVA) hydrogels, for coupling the bactericidal and wound healing properties maintaining the mechanical properties [18,19]. Sánchez-Aguinagalde et al. (2021) proposed the development of chitosan and PVA hydrogels reinforced with inorganic bioactive glass particles. Later authors indicated that the monitoring of the rheological properties of polymer-based matrices used as carriers is critically relevant during processing in order to select the most suitable final application [20].

In the present study, the solid residue recovered after the extraction of arnica was used as a raw material for the valorization of the residual phenolics and polysaccharide fractions. The conventional extraction processes using organic solvents or alkaline solutions were replaced by greener extraction stages. Both antioxidant and antitumoral properties were assessed, and based on the results, the formulation and rheological performance of novel chitosan or PVA gelled matrices loaded with arnica extract for external use was proposed.

## 2. Materials and Methods

### 2.1. Raw Material

Wild *Arnica montana* flowers were dried at room temperature for approximately two weeks and ground to obtain a suitable size (<0.5 cm) to continue with the extraction process. Commercial *Arnica montana* flowers (nutritional supplement, Disgadiet, Ourense, Spain), pre-treated as their wild counterparts and a marketable arnica (*Arnica montana* L. tincture, Soria Natural, Soria, Spain), were also used for comparative purposes.

### 2.2. Extraction Technologies

#### 2.2.1. Ethanolic Extraction

The ethanolic extracts was prepared from the raw material (ET) and from its commercial counterpart (CET). Extraction was performed with ethanol at 70% (from ethanol diluted with 96% of distilled water, Scharlab, Barcelona, Spain) having a solid:liquid ratio of 1:15 (*w*/*v*). The mixture was performed in a glass stirred tank at room temperature for 24 h. The liquid and solid phases were separated by filtration. The liquid phase was analyzed, and the solid phase (RS) was separated and used for further extraction of phenolic compounds.

#### 2.2.2. Ultrasound-Assisted Extraction

The solid phase of the wild raw material, after ethanolic extraction (RS-ET), was placed in an ultrasonic bath (P-selecta, Barcelona, Spain) using distilled water as a solvent and maintained at room temperature (25 °C). Two parallel ultrasound-assisted extraction (UAE) conditions were performed: (i) long extraction times (between 3 and 7 h and a solid:liquid ratio of 1:50, *w*/*v*) and (ii) short extraction times (between 20 and 120 min and a solid:liquid ratio of 1:20, *w*/*v*). The latter experiment was performed for comparative purposes in order to assess the total phenolics content and cell viability in four cell lines. The settings of the equipment were 1.5 A, 150 W, and 40 Hz. The suspension was separated by filtration, the phenolics content was analyzed in the liquid phase, and the solid phase was split for the next extraction process: pressurized hot water and microwave hydrodiffusion gravity-ultrasound assisted extraction.

#### 2.2.3. Pressurized Hot Water Extraction or Autohydrolysis

The solid sample from the ultrasound-assisted extraction process (RS-ET-UAE) was mixed with distilled water in a pressurized reactor (series 4842, Parr Instruments, Mobile, IL, USA) equipped with a stirred vessel (0.6 L). The liquid:solid ratio was 50:1 (*v*/*w*), and the reactor was heated to 120, 140, 160, 180, 200, and 220 °C following the fastest heating profile. When the reactor achieved the desired temperature, it was quickly cooled to room temperature. The suspension was filtered by vacuum, and the liquid phases were characterized.

#### 2.2.4. Microwave Hydrodiffusion Gravity-Ultrasound Assisted Extraction

The solid sample (10 g) from UAE (RS-ET-UAE) was introduced in an extraction vessel (1.5 L Pyrex) and irradiated at 100, 200, 300, 400, and 500 W for 20 min in the microwave extractor (NEOS-GR, Milestone Srl, Milan, Italy) operating at 2.45 GHz. An external sensor controlled the temperature of the sample. After this extraction by microwave hydrodiffusion and gravity (MHG) the solid samples were extracted with distilled water at a solid:liquid ratio 1:50 (*w*/*v*) in the ultrasonic bath (P-Selecta, Barcelona, Spain) for 5 h under 1.5 A, 150 W, and 40 KHz. This process was labeled as RS-ET-UAE-MHG. These experiments were carried out in duplicate, and the liquid phases separated by filtration were characterized.

### 2.3. Oligosaccharide Content

This fraction was analyzed in the liquid samples obtained from the tested extraction processes (RS-ET-UAE-AH and RS-ET-UAE-MHG-UAE). Samples were hydrolyzed with sulfuric acid at 4% (*v*/*v*), with the operation conditions: 20 min, 121 °C and 2 atm. The hydrolyzed samples were filtered through 0.45 μm cellulose acetate cartridges (Sartorius, Germany) and analyzed by high-performance liquid chromatography (HPLC, 1100 Agilent, Germany), provided by a refractive index detector. The column used was an Aminex HPX-87H (300 × 7.8 mm^2^) from BioRad (Hércules, CA, USA) working at 60 °C, the mobile phase was sulfuric acid (0.003 M), and the flow rate 0.6 mL/min. The standards used were glucose, galactose (Gal), xylose (Xyl), mannose (Man), rhamnose, arabinose, formic acid, acetic acid, and galacturonic acid from Sigma–Aldrich (Bratislava, Slovakia). Galactose, xylose, and mannose, eluting at the same retention time, were quantified as Gal + Xyl + Man.

### 2.4. Antioxidant Activity

The antioxidant activity of the samples was measured by ABTS radical cation (ABTS^+^) scavenging ([2,2-azinobis(3-ethyl-benzothiazoline-6-sulfonate)]) following the method developed by Re et al. [21]. The standard curve was carried out with Trolox (6-hydroxy-2,5,7,8-tetramethylchroman-2-carboxylic acid); therefore, this assay was expressed as the TEAC (Trolox equivalent antioxidant capacity) value. Briefly, diluted ABTS^+^ solution (1 mL), with an absorbance of 0.7 ± 0.1 at 734 nm, was added to sample (10 µL). After incubation at 30 °C for 6 min, the absorbance of the samples was measured at 734 nm using a spectrophotometer (Evolution 201, Thermo Scientific, Shanghai, China).

The reducing power assay was based on the reduction of Fe^3+^ to Fe^2+^ [22]. The liquid samples obtained from RS-ET-UAE-MHG-UAE and RS-ET-UAE-AH were assessed. Briefly, 1 mL of sample was placed in a test tube, 2.5 mL phosphate buffer (0.2 M and pH 6.6) and 2.5 mL of potassium ferricyanide (1%) were also added and mixed with a vortex. After incubation at 50 °C for 30 min, 2.5 mL of trichloroacetic acid (10%) were added, and then a centrifugation step for 10 min and 4500 rpm was needed. The supernatant was mixed with distilled water (1:1, *v*/*v*) and 0.5 mL of ferric chloride (0.1%, *w*/*v*). The standard curve was made with ascorbic acid, and the absorbance was read at 700 nm in an (Evolution 201, Thermo Scientific, Shanghai, China) spectrometer.

α,α–Diphenyl-β-picrylhydrazyl (DPPH) radical scavenging was evaluated for the samples obtained after RS-ET-UAE-MHG-UAE and RS-ET-UAE-AH. The protocol followed was: 2 mL of a 6 × 10^−5^ M methanolic solution of DPPH were added above 50 μL of a methanolic solution. The absorbance was read at 515 nm. All assays were performed at least in triplicate.

### 2.5. Total Phenolic Content

The total phenolic content was determined with the protocol proposed by Singleton and Rossi [23] using Folin–Ciocalteau reagent 1 N (1 mL), which was added with 20% sodium carbonate (2 mL) to the liquid samples (1 mL). After an incubation period at room temperature for 45 min in darkness, the absorbance was read at 730 nm using gallic acid (Sigma–Aldrich, Shanghai, China) as standard (spectrophotometer Evolution 201, Thermo Scientific, Shanghai, China).

### 2.6. Molar Mass Distribution

The molecular weight profiles of the liquid samples were assessed by high-performance size exclusion chromatography (HPSEC). The columns used were a pre-guard column PWX-guard (40 × 6 mm^2^) and the columns TSKGel G3000PW_XL_ and G2500PW_XL_ (300 × 7.8 mm^2^), both from Tosoh Bioscience (Stuttgart, Germany). A refractive index (RI) detector was used to obtain the chromatograms, with the operation conditions: 70 °C, Milli-Q water as the mobile phase, and 0.4 mL/min the flow rate. Dextrans from 1 kDa to 80 kDa were used as patterns.

### 2.7. Cytotoxicity

The cytotoxicity of the liquid samples obtained by the processes where MHG or AH were involved were evaluated on four cell lines from the European Collection of Cell Cultures: epithelial lung adenocarcinoma (A549), colon carcinoma (HCT-116), pancreatic adenocarcinoma (PSN1), and Caucasian human glioblastoma (T98G). Two cell lines, A549 and HCT-116, were grown in RPMI-1640 medium supplemented with 5% fetal bovine serum (both from Sigma), 1% penicillin/streptomycin (Hyclone, Thermo Scientific), and 1% L-glutamine (Sigma). A supplement of 10% fetal bovine serum, 1% L-glutamine, and 1% penicillin/streptomycin was required by PSN1 cells and T98G cells; later, these also required 1% sodium pyruvate and1% amino acid solution.

Thiazolyl blue tetrazolium bromide (MTT), from Sigma–Aldrich, was used to study the cell viability. The incubation of the cells was made with MTT solution (50 µL, 1 mg/mL) at 37 °C for 2 h with a humidified atmosphere of CO_2_ (5%). After this period, the MTT was removed, and 100 µL pure DMSO were added. The absorbance of the samples was read at 490 nm. The concentrations of the samples assessed were 25, 50, 250, and 500 µg/mL. The positive control was staurosporine from AQUAe collection (Biomar, León, Spain), while the negative control was the specific medium without cells. The cell growth inhibitions of the four cell lines were evaluated in the presence of staurosporine, with the IC_50_ value: 0.001 µg/mL for A549 cells, 0.005 µg/mL for HCT-116, 0.001 µg/mL for PNS1, and 0.001 µg/mL for T98G.

### 2.8. Formulation of Novel Topical Dressings

A first set of gelled matrices loaded with arnica extracts and tinctures was formulated with chitosan (10%, *w*/*w*) (Roig Farma, Barcelona, Spain), following the recommendations previously reported [24]. Briefly, the appropriate quantity of chitosan was dissolved in the dispersion medium used for the gelled matrix formation (i.e., CaCl_2_ (1%, *w*/*v*) and acetic acid (1%, *v*/*v*) stock solutions mixed in 1:3 volume ratio). All mixtures were stirred at 300 rpm for 60 min at room temperature.

A second set of gelled matrices was prepared with poly(vinyl alcohol) (PVA) (10%, *w*/*w*) (Alfa Aesar Chemicals, Tewksbury, MA, USA) based on earlier findings [19]. Concisely, the required amount of this synthetic biodegradable polymer was dispersed in deionized water at 90 °C for 4 h with constant stirring (300 rpm) and, subsequently, cooled (under stirring) to room temperature.

In both proposed gelled matrices, arnica extracts from ethanolic treatment, microwave, and ultrasound assistance (RS-ET-UAE-MHG-UAE) or pressurized hot water extraction under subcritical conditions (RS-ET-UAE-AH) were incorporated (15%, *w*/*w*) at room temperature. Commercial arnica tinctures were also assessed for comparative purposes. Before rheological testing, samples were cold stored overnight to favor full gels maturation. Finally, all systems were equilibrated at room temperature for 60 min before further analysis.

### 2.9. Rheology Testing

The rheology of selected gelled matrices was assessed at least in triplicate by means of steady and oscillatory shear measurements. Both experiments were performed in a controlled-stress rheometer (MCR 302, Paar Physica, Anton Paar, Graz, Austria) with a Peltier system for thermal control (±0.01 °C). A sandblasted parallel plate (25 mm diameter) was selected as measuring system. Gelled matrices were placed on the measuring system (1 mm gap), were covered with light paraffin oil and were rested for 5 min before rheology testing. In order to determine the viscous profiles and possible hysteresis effects, steady-shear flow curves were carried out by decreasing and then increasing the shear rate at 25 °C. The viscoelastic properties of the matrices were monitored at the same temperature, throughout the elastic (G′) and viscous (G′′) moduli. The linear viscoelastic region at above temperature was defined at <18 Pa for chitosan and <23 Pa for PVA systems using stress sweep measurements (from 0.1 to 100 Pa, 1 Hz). Subsequently, the oscillatory shear tests were measured at 2.5 Pa and 25 °C.

### 2.10. Statistical Analysis

Experimental data were assessed using one-factor analysis of variance, (ANOVA) employing statistical software (PASW Statistics v.22, IBM SPSS Statistics, New York, NY, USA). A post hoc Scheffé test was performed whenever differences between the means were identified using a 95% confidence interval (*p* < 0.05).

## 3. Results and Discussion

### 3.1. Extraction Yields, Chemical Features, and Antioxidant Properties

**Figure 1:**
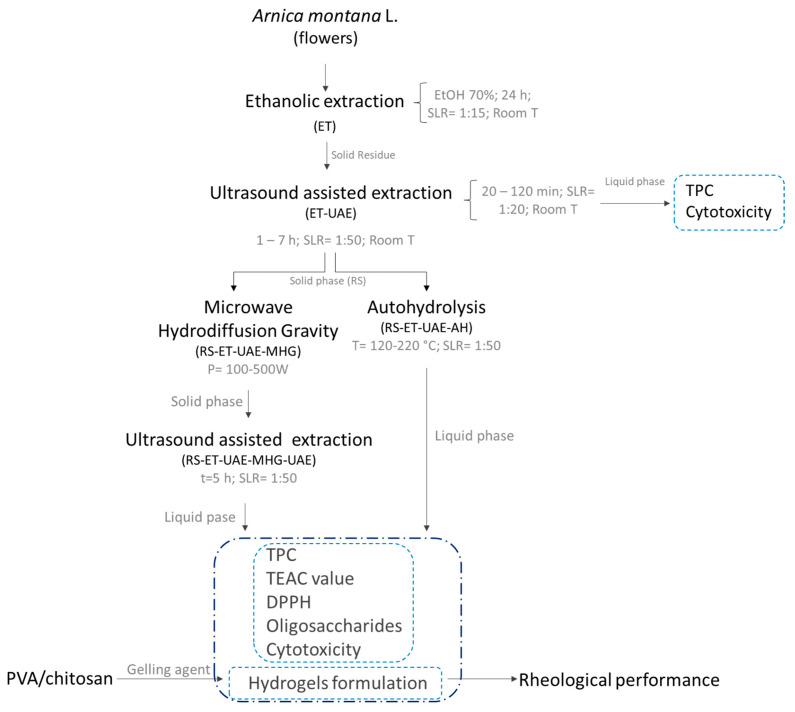
Diagram of the extraction process carried out to produce extracts from flowers of *Arnica montana* L. TPC, total phenolic compounds; PR, means reducing power; DPPH, free radical scavenging activity of the extract on 1,1-diphenyl-2-picryl-hydrazyl; TEAC value, Trolox equivalent antioxidant capacity.

The extraction process performed to obtain bioactive compounds from the raw material, *Arnica montana* L. flowers, is summarized in Figure 1. Following the scheme, the waste solids after ethanolic production of arnica were treated by ultrasound-assisted extraction (RS-ET-UAE). The extraction yield for the conventional ethanolic stage was 35.5% d.b., whereas the yield for the UAE water extraction to obtain the phenolics fraction exhibited an average value of 7%.

**Figure 2:**
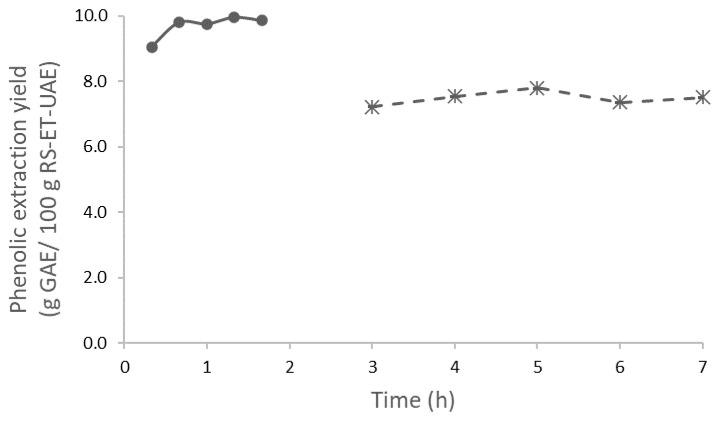
Total phenolics content for the US experiments using an LSR 50 (dashed line) and LSR 20 (line). Data represent *n* ≥ 3 and standard deviation (<5%).

Despite methanol and, in some cases, ultrasound assistance having been reported in different studies for their higher yields [10,13,14], in the present study only ethanol and water were used. The assisted process required shorter times than conventional extraction. The influence of the liquid-to-solid ratio (LSR) was also assessed. According to the trend shown in Figure 2 for the yield of phenolic content in the extracts, during the UAE (150 W, LSR 50) of the solids exhausted after conventional ethanolic extraction, the selected time was 5 h. The total phenolics extraction yield for this stage was 7.81% of the initial solids. On the other hand, the solids obtained after ethanolic extraction (RS-ET) were subjected to UAE, with the extraction conditions being different using an LSR 20 and short times compared to the previous process (20 versus 120 min). In this case, the results show the largest value at approximately 10% (Figure 2).

The solids remaining after RS-ET-UAE were further processed, either by microwave hydrodiffusion and gravity (MHG) and ultrasound-assisted water extraction (UAE) or by autohydrolysis (AH) (Figure 1). The combination MHG-UAE was established because MHG does not provide liquid extracts, probably due to the low water retention of the exhausted solids. However, it is expected that the microstructure can be altered, facilitating further solvent extraction [25]. Cavitation effects that take place during ultrasound treatment lead to swelling and hydration of the matrices, which are promoted in the presence of lower solid:liquid ratios.

**Figure 3:**
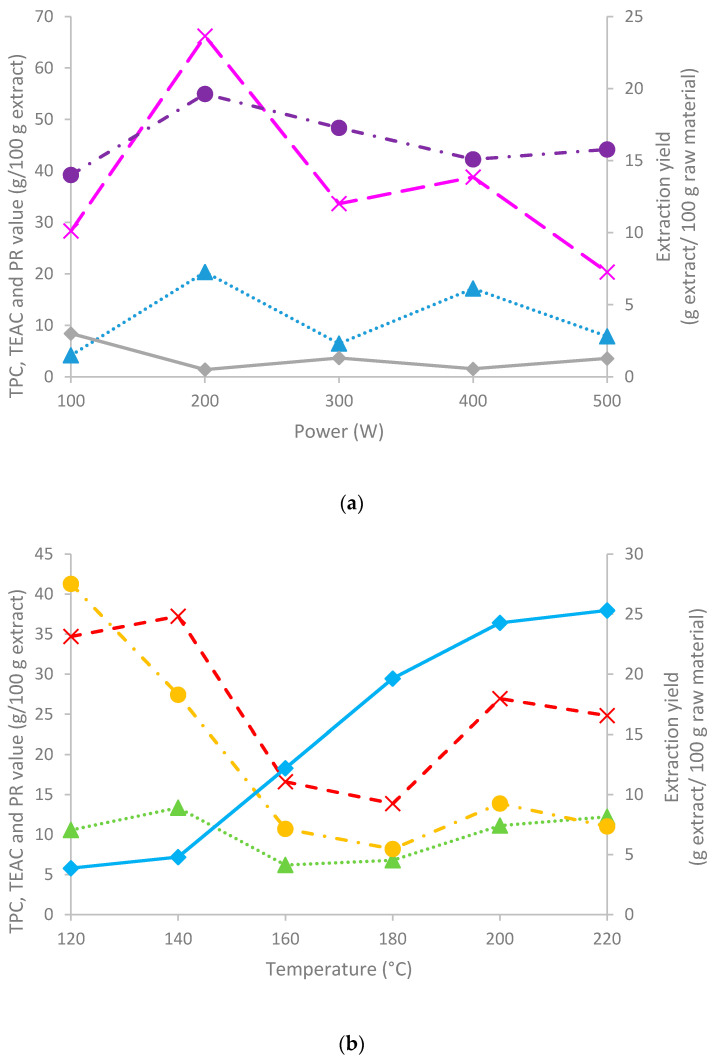
Influence of (**a**) the operation power during UAE on the microwave hydrodiffusion gravity-UAE stage, and the (**b**) the final heating temperature during autohydrolysis on the extraction yield (as g extract/100 g), total phenolics content (TPC), and TEAC value and reducing power (PR) of the extracts. Symbols: triangles (TPC), circle (PR), diamond (extraction yield), crosses (TEAC value). Data represent *n* ≥ 3; SD < 5%. Note here that the continuous lines correspond to the secondary axis.

The data in Figure 3a show the influence of the treatment power during MHG on the yields during UAE, with a maximum extraction yield at 100 W (3%). The extracts contained up to 20% phenolics, expressed as gallic acid equivalents, operating at 200 W, and the TEAC value showed a parallel behavior. In consistence with these results, the reducing power showed a maximum for the sample obtained by MHG at 200 W. The data in Figure 3b present the influence of the final heating temperature during autohydrolysis of the residual solids remaining after RS-ET-UAE-MHG. During autohydrolysis, a steady increase occurred from 140 to 200 °C with no further improvements at higher operation temperature. As expected, a similar trend in the phenolic contents and TEAC values were observed, with a maximum phenolic contents found at 140 and 200 °C or higher temperatures, with 12–14 g GAE/100 g extract. The ABTS antiradical capacity was maximum for the extracts produced during heating to 120 and 140 °C (no significant differences were observed). On the other hand, the reducing power exhibited the maximum value for the sample obtained at 120 °C. A DPPH assay was performed on the liquid samples obtained by RS-ET-UAE-MHG-UAE and RS-ET-UAE-AH. The results where MHG treatment was involved, showed a low inhibitory percentage; the maximum value was achieved for the sample obtained at 100 W (7.21%), whereas the minimum was observed for the sample obtained at 300 W. The liquid samples involving AH processing were also evaluated; in this case, all samples extracted from 140 to 220 °C showed an inhibitory percentage above 50%, obtaining a maximum inhibitory percentage (100%) for the sample obtained at 220 °C at the concentration of 0.60 g extract/100 g liquid sample. The minimum was observed for the sample obtained at 120 °C (46.85%), and for 140, 160, 180 and 200 °C, the inhibitory percentage values were 58.32%, 65.08%, 79.95%, and 97.61%, respectively. In this case, the EC_50_ was estimated for 140, 160, 180, 200, and 220 °C, with the values being 0.10, 0.22, 0.28, 0.25, and 0.25 g extract/100 g liquid sample.

These values were higher than those reported using direct ethanol extraction from the flowers [16,26]. These later authors reported an 18.4% extraction yield using 70% ethanol at room temperature for 8 h, and in the extracts they identified quercetin as the major component followed by rutin, apigenin, chlorogenic acid, caffeic acid, luteolin, gallic, and coumaric acids. Phenolic acids were also dominant in 80% of the methanolic extracts, mainly chlorogenic acid, and they also contained caffeic acid and protocatechuic acid [13]. The antiradical properties, determined as TEAC, ORAC (oxygen radical absorbance capacity), and 2,2-diphenyl-1-picrylhydrazyl free radical-scavenging, were correlated with the flavonoids and phenolic acids content [16]. Moreover, in another work where reducing antioxidant power was evaluated as FRAP, the authors found that *Arnica montana* L. was a powerful natural source of antioxidants [27]. Vidic and coauthors analyzed the antioxidant activity of four Asteraceae species, where the raw material of *Arnica montana* L. had highest antioxidant activity [28]. In the methanolic extracts, the presence of phenolic (quinic acids, inositol esters) and flavonoid (pyranone) compounds, which accounted for 98 mg GAE/g extract and 158 mg rutin eq/g extract, respectively, were responsible for their free radical scavenging capacity. Against superoxide, hydrogen peroxide, and nitric oxide radicals, the extracts were as active or more than standard antioxidants (BHT and ascorbic acid) [3]. Lower phenolic (24 mg GAE/g) and flavonoids (9 mg rutin eq/g) contents were observed in acetone extracts from exudates; these surface flavonoid aglycones were scutellarein 6-methyl ether (hispidulin), scutellarein 6,4′-dimethylether (pectolinarigenin), 6-OH luteolin 6-methyl ether, and kempferol-6-methyl ether [14]. These compounds showed antiradical properties but would be preferentially found in the first ethanolic extracts.

**Figure 4:**
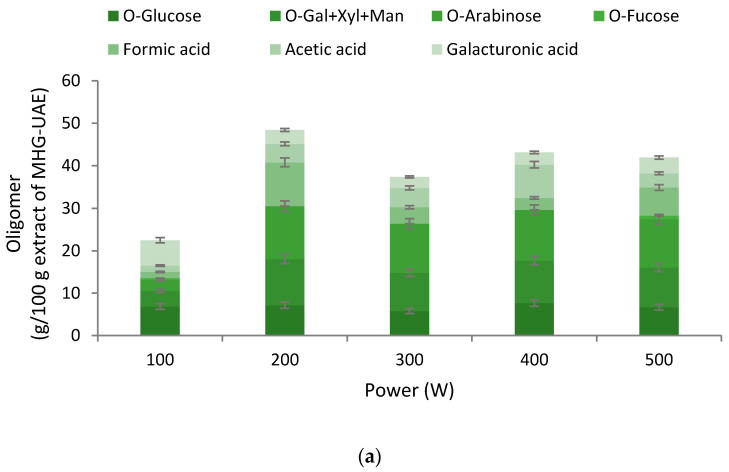

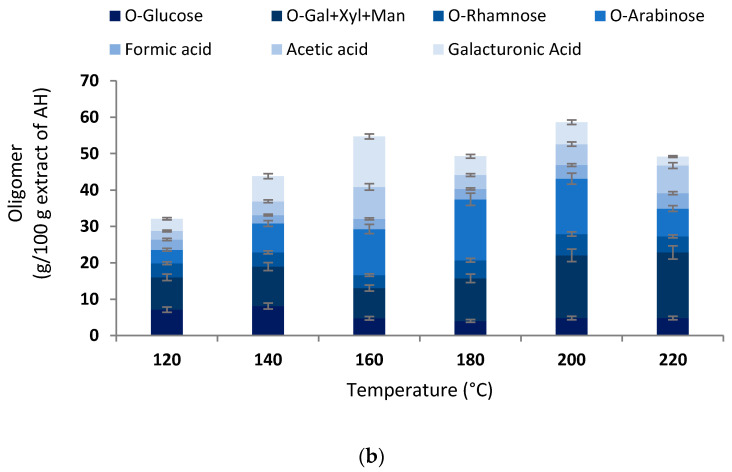
Influence of (**a**) the operation power during MHG on the UAE stage, and (**b**) the final heating temperature during autohydrolysis evaluated on the distribution of components in the oligosaccharide fraction. Data represent *n* ≥ 3 and standard deviation <5%. Note here that Gal + Xyl + Man corresponds to galactose+xylose+mannose.

The aqueous extracts obtained by UAE of the MHG treated solids (RS-ET-UAE-MHG-UAE) showed different saccharidic compositions depending on the treatment power. The maximum content was found at 200 W, with near to 40% recovery. The most abundant being arabinose, and the peak corresponding to galactose + xylose+mannose (Gal + Xyl + Man), which mostly corresponded to galactose (Figure 4a).

The aqueous extracts obtained after autohydrolysis or subcritical water processing operating in non-isothermal mode up to maximal temperatures in the range 120–220 °C presented very low content in monomers, and most of the saccharides were found as oligomers. The major recovery of the oligosaccharides was found at 200 °C, and glucose content was significantly lowered when the temperature increased up to 140 °C. The extraction of arabinose significantly increased until 180 °C, and then the value decreased. On the other hand, in treatments at higher temperature, the most abundant was the sum of galactose+xylose+mannose, obtaining a value of 18% at 220 °C (Figure 4b).

**Figure 5:**
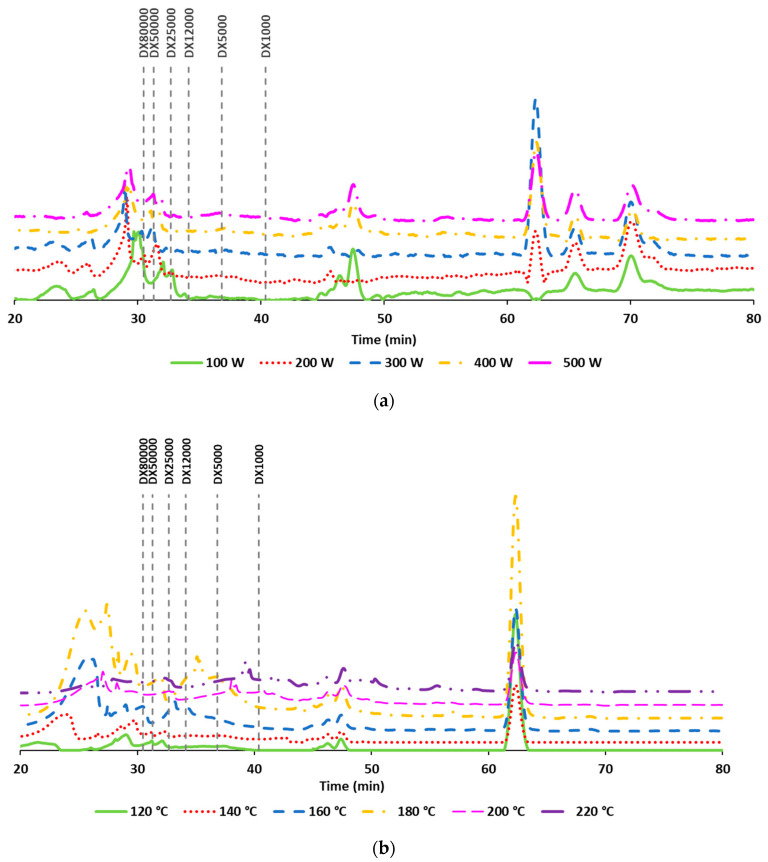
Molar mass distribution of the liquid samples obtained by microwave hydrodiffusion gravity-UAE at different powers (**a**) and autohydrolysis (AH) at different final heating temperatures (**b**).

Figure 5 shows the HPSEC profiles of the extracts. Regardless, the extraction power, where MHG-UAE were involved, produced a number of low molecular weight compounds (under 1 kDa) and a group of peaks corresponding to compounds of more than 80 kDa. The use of higher power seemed to cause an increase in one of the lower molecular weight compounds and a decrease in the content of the higher Mw compounds (Figure 5a). The extracts obtained under AH treatment showed a maximum content of the lower molecular weight compounds during heating to 180 °C. Whereas during operation at 160 °C, a wide peak corresponding to 20 kDa was observed; heating at 180 °C produced a wide peak at 10 kDa.

Other authors reported that the product obtained from the flowers by alkaline extraction, solvent extraction, and dialysis yielded 5.7% extract with two peaks of 9 and 3.5 kDa and contained 26% carbohydrates, 21% phenolics, 12% uronic acids, and 1% protein and mineral fractions [2]. They found that the major saccharide constituents were arabinose (36%), galactose (28%), glucose (18%), and rhamnose (13%), whereas xylose and mannose were found in lower amounts, and arabinogalactan and rhamnogalacturonan were the dominant features.

### 3.2. Cytotoxicity

**Figure 6:**
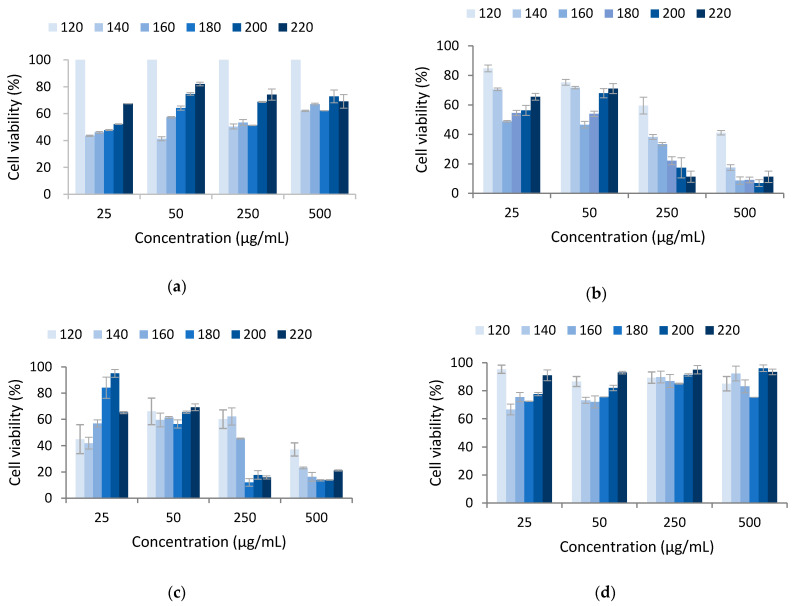
Cell viability of selected human tumoral cells in contact with the liquid phases obtained after autohydrolysis at different final temperatures from 120 to 220 °C for the cell lines: A549 (**a**), HCT-116 (**b**), PSN1 (**c**), and TG98 (**d**). When standard deviations were lower than the symbol size, it was not plotted in the corresponding graph.

The extracts obtained after AH processing of the exhausted solids after RS-ET-UAE showed cytotoxic properties against HCT-116 and PSN1 cells, with an EC_50_ of 250 µg/mL for those produced while heating to 160 °C and higher autohydrolysis temperatures (Figure 6).

No clear trend in the power during MHG-UAE was observed, and the EC_50_ could not be calculated, except for A548 cells, which at 200 and 500 W was approximately 50 µg/mL, and for HCT-116 the extracts produced at 200 W showed 250 µg/mL and against PSN-1 cells, the extracts obtained at 400 W showed 5 µg/mL (Table 1).

The results of the extraction using ultrasound at short times are shown in Table 2. In this case, no clear effect was observed; however, the A549 and TG98 cell lines did not show cell inhibition, and the HCT-116 cell line exhibited a cell viability of 38% at 250 µg/mL of the extract obtained after 120 min of extraction (LSR 20). On the other hand, the TG98 cell line also approximately revealed a 38% cell viability in contact with 250 µg/mL of the extract obtained after 100 min of extraction as well as a 31% cell viability using the largest concentration of extract, 500 µg/mL. Similarly, the phenolic–carbohydrate arnica complex extracted with alkaline treatment and fractionated by solvent extraction and dialysis showed a slight cytotoxic effect [2].

### 3.3. Rheology of Potential Topic Dressings

**Figure 7:**
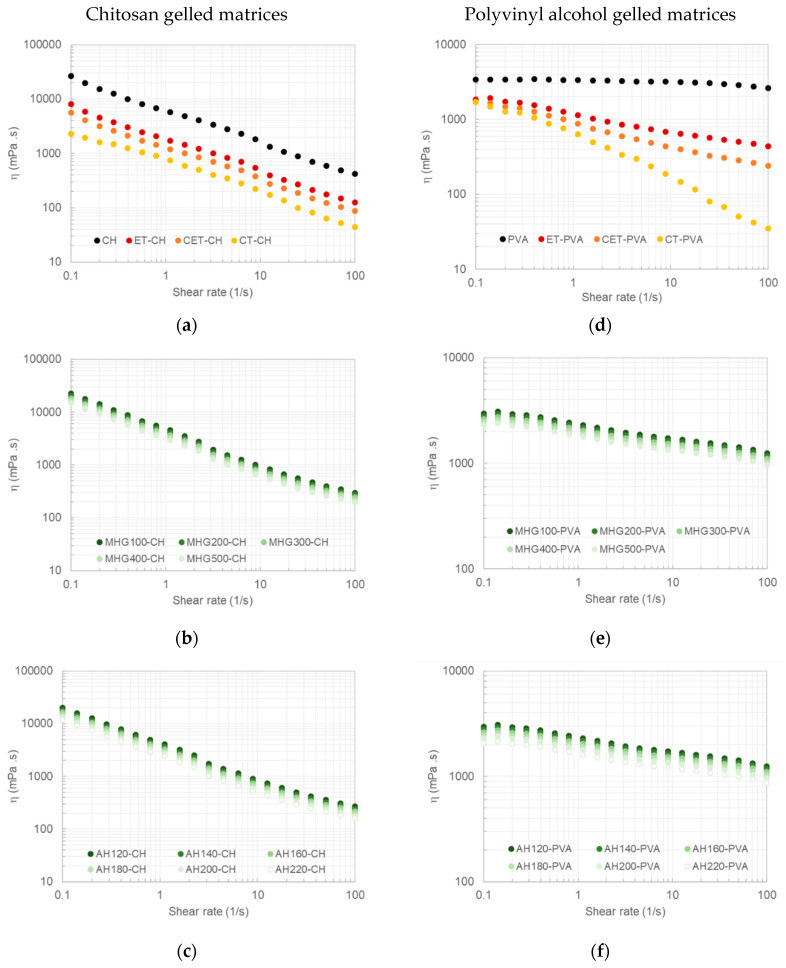
Effect of the processing conditions on the viscous profiles of gelled matrices formulated with chitosan (CH, left column) or polyvinyl alcohol (PVA, right column) loaded with ethanolic arnica extracts (ET, CETs) and commercial tinctures (CTs) (**a**,**d**) as well as with arnica extracts obtained after MHG-UAE treatments at different powers (**b**,**e**) or AH processing at tested final temperatures (**c**,**f**). Standard deviations were lower than 2.5% in this and subsequent plots.

The topical use of the extracts obtained was proposed, because the phenolics fraction could contribute to the anti-inflammatory properties and the oligomeric fractions to the gel’s strength [4]. In order to assess this possibility, the influence of the addition of the obtained extracts on the formulation of two gel types was evaluated and compared to both the ethanolic extracts (prepared and commercial). Figure 7 shows the viscous profiles of gelled matrices formulated with chitosan (CH) (a, b, c) and poly(vinyl alcohol) (PVA) (d, e, f) at 10% loaded with a number of arnica tinctures at 15%. Samples prepared with both polymers exhibited shear-thinning behavior, with decreasing apparent viscosity as the shear rate increased. In all cases, the drop in viscosity over the tested shear rate range was higher for chitosan matrices (about two decades) compared to PVA (about 1.2-fold). The incorporation of arnica tinctures did not noticeable modify the shear-thinning behavior of chitosan systems, whereas a remarkable effect was noticed on those made with PVA. The shear-thinning of gelled matrices containing arnica extracts recovered after the MHG-US or AH stages and slightly increased (approximately twofold) when compared with those loaded with ethanolic extracts (ET, CET) (approximately fourfold) and commercial tinctures (CTs) (approximately 50-fold). At a fixed shear rate, the apparent viscosity magnitude of both gelled matrices decreased in the presence of arnica extracts according to the following trend: CT > CET > ET-UAE > AH > MHG-UAE. Note here that the slight effects on the apparent viscosity values were identified for the irradiation power using during the MHG stage or the final temperatures selected during the AH stage. In general, a significantly increase in both parameters led to a slight decrease in the apparent viscous profiles. The viscous performance of polymeric gelled matrices proposed in this work were consistent with those previously reported for chitosan gel formulations recommended for healing burn wound injuries [24]. According to this study, proposed matrices could be properly spread out on applying shear for topic dressings. A slight increase in shear thinning behavior in the presence of extracted arnica could be advantageous, since the gel matrix would become more fluid while it is being spread over the injured skin, leading to a less painful and easier application.

**Figure 8:**
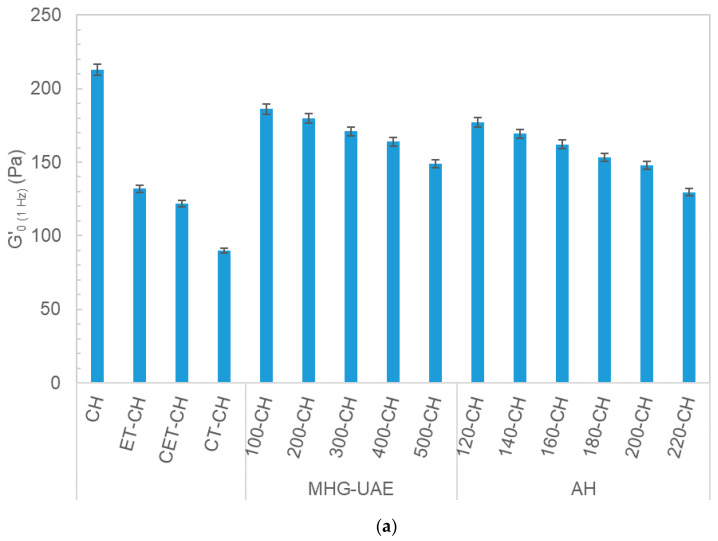

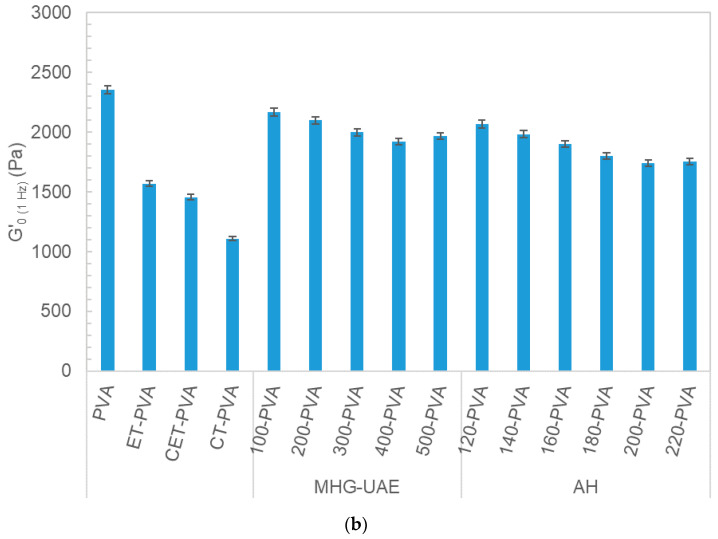
Impact of the processing treatments on the viscoelastic features of the gelled matrices formulated with chitosan (CH, (**a**)) or polyvinyl alcohol (PVA, (**b**)) incorporated with ethanolic arnica extracts (ET, CETs) and commercial tinctures (CTs) as well as with arnica extracts after the MHG-UAE stage at different powers or AH processing at tested final temperatures.

An overview of the viscoelastic performance of above gelled matrices is displayed in Figure 8. As all systems exhibited typical gel behavior, G′0 (1 Hz) was selected to show the effect of the different tested arnica systems on the mechanical features of the prepared gelled matrices. In all cases, PVA gelled matrices showed stronger viscoelastic properties (approximately 10-fold) when compared with chitosan ones loaded with the same arnica extracts. The relative impact of the arnica on the viscoelastic values was higher in the chitosan matrices, with a higher drop at the highest MHG irradiation powers or AH final temperatures. This behavior is also consistent with the results previously found for other PVA-bioactive natural products gels [19], where the addition of natural products to the PVA matrix did not cause significant variation in the mechanical values. The latter authors explained that it is known that antioxidant addition within PVA gelled matrices can commonly drop the viscoelastic moduli. The magnitude of the viscoelastic modulus experimentally determined in this work was in the range of those of other mixtures of polymer-based systems from the literature, considered mechanically suitable for topical use [19,29]. Moreover, the authors also indicated the adequate ability of this kind of gelled matrices with different natural products to deliver phenolic compounds. Compared with the oligomeric results obtained by HPSEC, it seems that mild processing conditions involved oligomeric fractions with higher average molecular weights and higher viscous and elastic features of the corresponding gelling matrices. Overall, these outcomes support the hypothesis that the interplay between biopolymers and oligomers-OH groups, create more stable junction zones that are promoted in the presence of high molecular weight oligomers [30].

## 4. Conclusions

Valorization of the fractions remaining in the solids after ethanolic extraction from *Arnica montana* flowers was proposed following a sequence of stages using green solvents with ultrasound, microwave, and pressurized extraction. The sequence of stages allowed the extraction of approximately 28% total phenolics. The extracts contained up to 50% oligosaccharides that could contribute to the observed gelling properties of the films developed for topical use. Rheological findings suggest that proposed chitosan and polyvinyl alcohol gelled matrices could be attractive alternatives for the development of topical dressings with healthy properties in the absence of water syneresis. As a future trend, it should be interesting to continue the assessment of the sequential processes based on green technologies in order to extract compounds with biologic potential. A detailed chemical characterization and bioactive and functional performance of the product developed for different applications is planned for future works.

## Figures and Tables

**Table 1:** Maximum values of cell viability of human tumoral cells contacted with the liquid phases obtained after microwave hydrodiffusion gravity-ultrasound assisted extraction stage, where the powers of microwave were from 100 to 500 W and the ultrasound-assisted extraction was alike for all the residue samples (5 h, 1.5 A, 150 W, and 40 Hz). The TG98 cell line did not shows cell inhibition. Data represent the mean ± SEM (*n* = 3).

	Cell Viability (%) of Extracts Obtained at Different Microwave Powers (Extract Concentration; µg/mL)				
Cell Line	100 W	200 W	300 W	400 W	500 W
A548	78.3 ± 1.5; (50)	46.7 ± 0.6; (50)	56.8 ± 1.6; (50)	58.5 ± 0.3; (25)	42.4 ± 1.3; (50)
HCT-116	63.8 ± 5.1; (25)	49.2 ± 7.4; (250)	52.9 ± 2.4; (500)	65.3 ± 5.0; (500)	57.0 ± 16.2; (50)
PSN1	69.4 ± 3.6; (25)	69.9 ± 2.6; (25)	53.0 ± 4.5; (25)	50.4 ± 0.5; (25)	65.5 ± 6.7; (25)

**Table 2:** Cell viability of human tumoral cells contacted with the liquid phases obtained after ultrasound-assisted extraction (UAE) from 20 to 120 min. The A549 and TG98 cell lines did not show cell inhibition. Data represent the mean ± SEM (*n* = 3).

	Cell Viability (%) Obtained at Different Extraction Times (Extract Concentration in µg/mL)					
Cell Line	20 min	40 min	60 min	80 min	100 min	120 min
HCT-116	76.1 ± 5.3; (500)	49.3 ± 3.1; (500)	59.1 ± 5.9; (500)	49.8 ± 3.8; (500)	50.3 ± 2.2; (500)	38.7 ± 2.4; (250)
PSN1	55.6 ± 1.4; (250)	31.2 ± 0.8; (500)	43.1 ± 1.1; (500)	73.9 ± 1.9; (500)	37.6 ± 1.0; (250)	43.9 ± 1.1; (250)

## Data Availability

Data were reported in the main document.
